# Monitoring clinical quality in rare disease services – experience in England

**DOI:** 10.1186/1750-1172-3-23

**Published:** 2008-09-15

**Authors:** Thomas D Kenny, Edmund G Jessop, William H Gutteridge

**Affiliations:** 1 National Commissioning Group, Southside, 105 Victoria Street, London, UK

## Abstract

After some well-publicised problems with paediatric cardiac surgery, there has been great interest in England in monitoring clinical quality in specialised medical services. The National Commissioning Group plans, funds and monitors a set of highly specialised services for the National Health Service in England. We have developed systems for monitoring clinical quality that perform two interrelated but distinct functions: performance measurement and performance improvement. The aim is to collect information on all patients seen during each year – a 100% consecutive case series. Generally, there is no conceptual difficulty identifying an appropriate outcome for surgical interventions: the indication for surgery usually defines the outcome to monitor. This is not so for the medical and psychiatric services, where the relevant outcome to monitor is sometimes not obvious. There are a number of problems in interpreting, and acting on, outcome data for rare conditions and treatments. These problems include statistical problems due to small numbers, the need to risk adjust data and coding problems.

## Introduction

After some well-publicised problems with paediatric cardiac surgery in Bristol [1], there has been great interest in England in monitoring clinical quality in specialised medical services. The National Commissioning Group [2] plans, funds and monitors a set of highly specialised services for the National Health Service in England. These services fall into three groups: surgical interventions; medical or psychiatric care; and diagnostic procedures. We have developed a system of monitoring these services which we describe and discuss in this paper.

In developing and implementing systems for monitoring clinical quality we have appreciated that they perform two interrelated but distinct functions: performance measurement and performance improvement. The purpose of performance measurement is to ensure that all the centres involved in providing a service are offering equivalent quality. Promoting performance improvement through review of outcome data is equally important.

In rare disease services we are dealing with complex multi-component interventions. The number of patients is so small that learning is unlikely to occur spontaneously [3,4]. This is true even if we network at a health system level. Therefore, we need mechanisms to actively drive learning [3,4]. Learning will not happen passively, particularly in isolated single centres. The aim of our process for monitoring outcomes is to contribute to developing expertise and quality.

## Methods

For the surgical and medical group of services, the aim is to collect information on all patients seen during the year – a 100% consecutive case series. For each service, specific outcome measures are chosen based on the characteristics of the disease and the service. The main measures are set out in Tables 1, 2, 3. When collecting outcome data we use the following definitions:-

**Table 1:** Clinical outcomes used as indicators to assess the performance of surgical services

**Condition or Intervention**	**Outcome measure**
Bladder exstrophy	Immediate complications
Bone cancer	Three-year local recurrence
Adult Ventricular Assist Devices (Bridge to heart transplant)	Survival
Child Ventricular Assist Devices/Extracorporeal Membrane Oxygenation (ECMO) (Bridge to heart transplant)	Deaths
Complex tracheal disease	Deaths, Re-intervention, Stents, Repeated outpatient attendances, Repeated inpatient attendances.
Craniofacial surgery	Appropriate measure not clear
Extracorporeal Membrane Oxygenation (ECMO) (adult)	Survival without severe disability at 6 months
Extracorporeal Membrane Oxygenation (ECMO) (neonate & infants, children)	Survival to discharge
Malformation of female genital tract	Research study published: 20-year outcome on sexual function [22].
Ocular oncology	Loss of eye (primary and secondary enucleation); Local recurrence.
Oculo-odonto-kerato-prosthesis	Visual acuity
Pseudomyxoma of peritoneum	Complications, median survival
Pulmonary thromboendarterectomy	In-hospital mortality
Retinoblastoma	Deaths; Loss of eye (primary and secondary enucleation); Metastasis.
Severe Combined Immunodeficiency	Deaths
Transplant – heart	Mortality at 30 days after transplant; Survival one and five years after transplant
Transplant – liver	Mortality at 90 days after transplant; Survival five years after transplant
Transplant – lung	Mortality at 90 days after transplant; Survival one and five years after transplant
Transplant – pancreas	Mortality at 90 days after transplant; Survival five years after transplant
Transplant – small bowel	Mortality at 90 days after transplant; Survival five years after transplant
Vein of Galen malformation	Deaths and disability

**Table 2:** Clinical outcomes used as indicators to assess the performance of medical treatment and mental health

**Condition or Service**	**Outcome measure**
Alstrom syndrome	Appropriate measure not clear, there are biochemical markers of quality of care.
Chorioncarcinoma	Deaths
Epidermoloysis bullosa	Appropriate measure not clear, there are biochemical markers of quality of care.
Intestinal failure	Annual mortality
Lysosomal storage disorders	Quality of life scales currently in development
Mental health service for deaf adolescents	Global function score
Obsessive compulsive disorder	Yale-Brown Obsessive Compulsive Score
Paediatric liver disease	Heterogeneous category: five marker conditions are under consideration; Hepatoblastoma, Biliary atresia, Tyrosinaemia, Autoimmune sclerosing cholangitis, Bile acid disease. For all of these survival is the outcome indicator.
Paediatric pulmonary hypertension	Survival
Persistent Hyperinsulinemic hypoglycaemia of infancy (PHHI)	Survival and disability
Secure forensic mental health service: for young people	Health of the Nation Outcome Scales for Children and Adolescents (HONOSCA) and; Health of the Nation Outcome Scales (HONOS) Secure plus global function

**Table 3:** Clinical outcomes used as indicators to assess the performance of diagnostics

**Condition**	**Outcome measure**
Amyloidosis	External Quality Assurance Services (EQAS) plus external inspection
Mitochondrial disorders	External Quality Assurance Services (EQAS) plus external inspection
Ophthalmic pathology	External Quality Assurance Services (EQAS) plus external inspection to be arranged
Primary ciliary dyskinesia	To be determined
Rare neuromuscular disorder	External Quality Assurance Services (EQAS) plus external inspection

• Deaths – actual number of patients who died within a set time period after the intervention *e.g*. three months.

• Survival – number and/or % of patients surviving within a set time period after the intervention

• Mortality – number and/or % of patients dying within a set time period after the intervention

• Median survival – the time period that 50% of patients survive after the intervention

The data collected must be important to the centres themselves. They must regard data as a means to ensure they are aware of their own performance related to the national service as a whole. In practice, services usually become aware of an unexpected run of worse outcomes before data is formally analysed, but at a minimum the data collection is necessary in case a centre does not recognise a worsening of results. Collection and analysis of outcome over time is particularly important to put into context newly arisen concerns about performance at an individual centre.

We do not impose outcome measures unilaterally, but agree them with clinicians who provide the services. Imposition of measures without agreement may impair the constructive relationship between commissioners and the services, reduce compliance, become a mechanical exercise resented by the services and possibly lead to perverse incentives, such as not treating high-risk patients. Agreement on outcomes also involves reassurance about how we will interpret and use data on small numbers of patients. A sceptic might argue that it is harmful to collect outcome data because of the risk of misinterpretation. On the other hand, data may, in spite of all its limitations, suggest a possible difference in outcomes that needs further exploration. The important thing is that the services trust us to use the data sensibly.

In surgical services, the commonest outcome monitored is survival, though sometimes another measure is more appropriate: for example, visual acuity after an eye procedure, or local recurrence after surgery for bone sarcoma.

**Figure 1:**
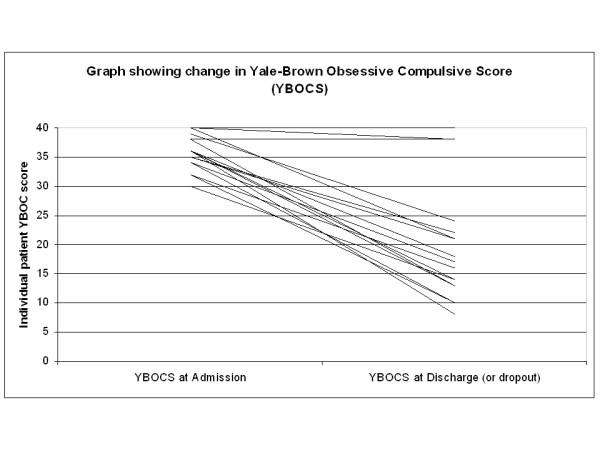
**Graph showing change in YBOC score**. Patients with OCD/BDD admitted to Heather Ward, South West London and St George's Mental Health NHS Trust April 2007 to April 2008, with YBOC score at admission and at discharge or dropout.

Generally, there is no conceptual difficulty identifying an appropriate outcome for surgical interventions: the indication for surgery usually defines the outcome to monitor. This is not so for the medical and psychiatric services, where the relevant outcome to monitor is sometimes not obvious. In one or two services, a well-validated scale is available to track improvement in the patient's disease. Thus the Yale-Brown Obsessive Compulsive Score (YBOCS) [5-7] meets this requirement. Before and after treatment scores can be used to monitor the effectiveness of treatment in the service for obsessive compulsive disorder (Figure 1).

Although all services aim to improve quality of life for patients, two problems arise where no disease-specific validated scale exists. Firstly, a generic measure may not be sensitive to the type of change one can realistically expect, even from a high quality service, in a condition with no option for disease modifying treatment. Epidermolysis bullosa provides an example of this. Secondly, generic quality of life scores are expensive (many are protected by copyright) and time consuming to administer. Hence biochemical proxies, though imperfect, may have to do duty as measures of the quality of medical care. Indices of glycaemic control in Alstrom syndrome are an example [8,9].

In the diagnostic services, the 'outcome' is a correct diagnosis. But since these are the national diagnostic centres, the diagnosis they make is, in one sense, the 'correct' diagnosis by definition. Hence, our focus in these services is to ensure participation in external quality assurance (EQA) schemes, together with a laboratory inspection system.

So far we have discussed outcome in patients who have attended a specialised service. But in a national system of care, we need also to check that the centres are serving the entire population of the country, and not just those who live nearby, since distance between referrer and specialised centre can be a barrier to care [10,11]. To examine this we use geo-spatial maps created in MapInfo^©^.

## Results

Within this section we will use, as examples, data from several services we commission. Outcome data collection and analysis should also be used more broadly as part of joint national audit involving all the centres for a particular service. The centres involved in each service are expected to meet as a whole at least once per year in a formal audit day. Here they present individual centre and joint national audits, share experiences and discuss the outcome data on all patients seen during the most recent year (or years if the numbers are very small) – the 100% consecutive case series (Figure 1 and Table 4). In this process, centres compare outcomes, discuss apparent differences and learn from each other (Table 5). This is vital in services involving small numbers of patients and important but rarely seen complications [3,4,12].

**Table 4:** Visual acuity results after Osteo-Odonto-Keratoprosthesis (OOKP) surgery

**Result**	**Number of Patients**
> 6/12	19
6/18 – 6/60	9
< 6/60	2
No Improvement	6

Source: [23]

**Table 5:** Patient mortality by liver transplant centre for all adult patients between 1st March 1994 and 31st March 2006 in England.

**Centre**	**Total**	**90-day mortality % (95% CI)**	**5-Year mortality % (95% CI)**
A	342	9.1 (6.5 – 12.6)	27.0 (22.2 – 32.5)
B	908	10.8 (9.0 – 13.0)	31.2 (27.8 – 34.9)
C	663	6.5 (4.9 – 8.7)	24.8 (21.3 – 28.7)
D	616	11.5 (9.3 – 14.3)	30.1 (26.4 – 34.2)
E	1247	6.1 (4.9 – 7.6)	26.9 (24.1 – 30.0)
F	1268	10.6 (9.1 – 12.5)	24.5 (22.1 – 27.2)

Source: [24]

Patient mortality by transplant centre up to 5 years post liver transplant for all adult patients who received a first liver transplant as elective between 1^st ^March 1994 and 31^st ^March 2006 in England

**Figure 2:**
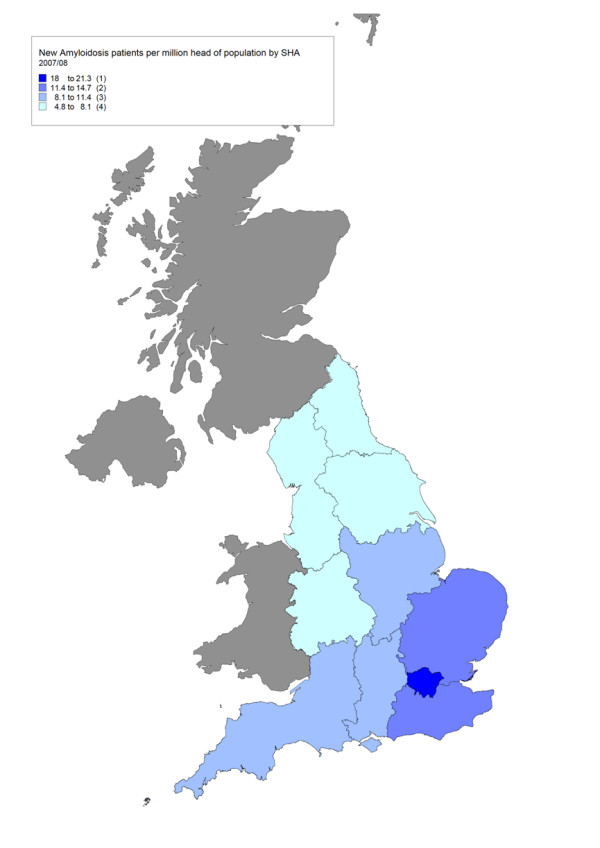
**Map of new amyloid referrals per 1 million population by English Strategic Health Authority 2007–08**. This map of new amyloid referrals to the specialist service covers England only and not Scotland, Northern Ireland or Wales.

**Figure 3:**
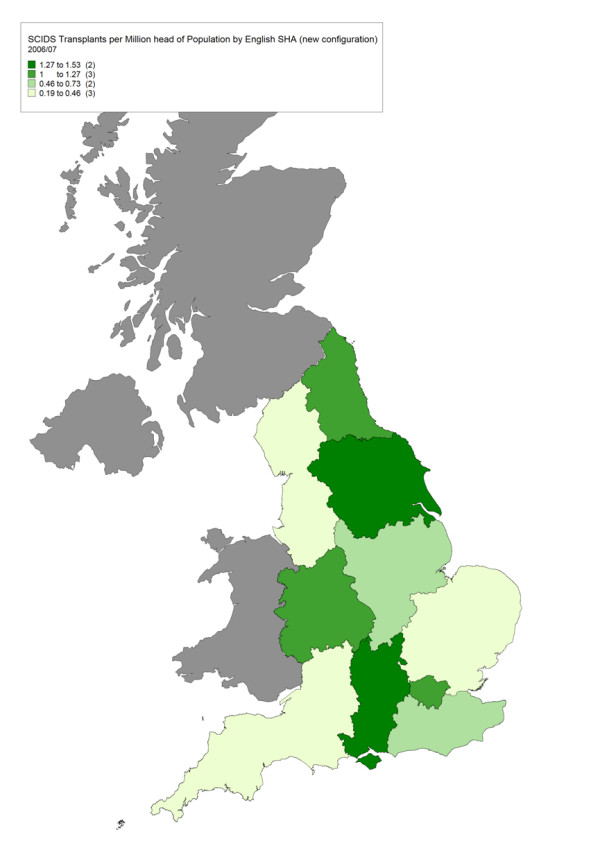
**Map of Transplants for Severe Combined Immunodeficiency per 1 million population by English Strategic Health Authority 2006–07**. This map of transplants for severe combined immunodeficiency covers England only and not Scotland, Northern Ireland or Wales.

The true prevalence and spatial distribution of many very rare diseases is not known. When examining the geographical access to services, our working assumption is that case ascertainment is likely to be most complete near a specialised centre where awareness is high (Figure 2); and that low rates distant from the centre are evidence of poor access until proven otherwise (Figure 3).

Poor access may be a result of travel difficulties or failure by local clinicians to recognise patients in need of highly specialised care. Where a clear geographical inequity in service use is found, options for action include the development of collaborative arrangements with a local hospital, the commissioning of a new specialised centre or, where appropriate, increasing awareness in non-referring regions.

## Discussion

There are a number of problems in interpreting, and acting on, outcome data for rare conditions and treatments. The first group of issues are statistical. For some services, even though the National Health Service in England has a population base of 50 million patients, there are too few patients for meaningful statistical analysis. Examples include congenital hyperinsulinism, bladder exstrophy and the Vein of Galen malformation.

A further problem, particularly when centres are compared with each other, is interpretation of crude outcome data that are not adjusted for risk. This is especially difficult for conditions such as retinoblastoma, where on average there are only 50 new patients in the UK each year, two types – bilateral and unilateral – with different underlying genetics, and four clinical grades relevant to treatment and preserving vision.

Where numerical analysis is possible, other issues arise. One example is the alert threshold at which further investigation is needed. This could be a relative or an absolute threshold, for example a 10% difference in death rates or an excess of 5 deaths above expectation. Setting such thresholds is arbitrary and needs to be agreed with the services. We have little experience of making these judgements for outcomes other than mortality – it is not clear what difference in quality of life outcomes, for example, should trigger further action.

In services with more than one centre we look to see that there are no statistically significant differences in outcome between the centres (Table 5). For services with one centre we can determine whether or not the centre meets an agreed outcome threshold. In the Osteo-Odonto-Keratoprosthesis (OOKP) surgery service (Table 4) the agreed threshold is a post-operative visual acuity of 6/12 or better in at least 50% of patients.

'Expectation' may be set against the unit's own historic performance, using a sequential test analysis such as the variable life adjusted display (VLAD) [13,14], or the comparator may be based on the overall performance of all units providing the same service [15]. In practice these analyses are performed only for transplant services where the volume of data is large enough, for example, for adult liver and cardiothoracic transplant. These analyses are performed by our colleagues at the national transplant organisation [16] and by an academic unit within the Royal College of Surgeons [17]. Whatever expectation is chosen, problems of Type I and Type II errors remain in judging the true meaning of any statistically significant difference from expectation. There are other statistical techniques for examining rare adverse events that may be useful, such as *g*-type statistical control charts [18,19], but as yet we have no experience of using these.

Ideally, we would like also to compare results at UK centres with the best international centres. This is rarely possible. Publications in the international literature are almost always focused on selected subsets of the centre's caseload rather than a 100% consecutive case series. Furthermore, problems of comparability remain because the centres in England, unlike those elsewhere, have responsibility for all cases arising in England whereas specialised centres in other health systems almost always have selected caseloads. In addition, case-mix and threshold for intervention may not be the same.

Our generic approach for investigating apparent change or differences in outcome is to initiate a case note audit. If problems are detected, the centres may change their clinical practice appropriately. For example a transplant centre may change its donor or recipient eligibility criteria; or a surgical service may change its anticoagulant regime. If problems persist and are clearly intractable, then we may as a last resort request or require a centre to stop providing the service.

Mapping patient attendance at specialised centres has been discussed above. More properly when we consider mapping data, we should measure, not just access to the specialised service, but health outcomes. This potentially takes two forms. First is the relationship between distance to treatment centre and health outcome. We have not yet used the geospatial data for this purpose. The second way to look at health outcomes is to examine data for the whole population of England, whether or not the specialised service has been accessed. This is only feasible where mortality, or perhaps median age at death, is a relevant outcome. Median age at death has been used, for example, to judge the success of national health systems in caring for people with cystic fibrosis [20].

Where geographical inequities persist, there are a number of possible actions. These include educational initiatives, development of outreach or shared care arrangements between local services and the specialist centre, and establishing a new centre.

For the very rare conditions in the English national commissioning system, coding is a major obstacle to retrieving the necessary mortality data. Almost none have their own ICD code, but are included in larger categories (*e.g*. ICD-10 E75.2 includes Fabry's, Gaucher, Krabbe, Niemann-Pick, Farber's syndromes, metachromatic leukodystrophy, sulfatase deficiency). Choriocarcinoma is an exception with the ICD-10 code C58, but the number of deaths each year is so small (one or two) that coding errors become an important concern. Consequently, we have to ask the services to provide activity data themselves. Routine hospital data systems are unreliable because of the lack of codes for highly specialised treatments.

Finally, we regularly monitor patients' compliments and complaints about services. The UK literature suggests patients do not reliably judge the technical quality of their care against objective standards [21]. However, each centre is expected to carry out a patient satisfaction survey every three years, because we believe this gives an indication of the quality of the process of clinical care.

## Competing interests

The authors declare that they have no competing interests.

## Authors' contributions

TDK participated in the design and coordination of this article and drafted the manuscript. EGJ conceived of the article, and participated in its design and helped to draft the manuscript. WHG participated in drafting and revising the manuscript. All authors read and approved the final manuscript.
